# Comparison Between Simulated and Actual Unilateral Hearing in Sequentially Implanted Cochlear Implant Users, a Cohort Study

**DOI:** 10.3389/fsurg.2019.00024

**Published:** 2019-05-08

**Authors:** Alice van Zon, Yvette E. Smulders, Veronique J. C. Kraaijenga, Gijsbert A. van Zanten, Robert J. Stokroos, Inge Stegeman

**Affiliations:** Department of Otorhinolaryngology, Head and Neck Surgery, University Medical Center Utrecht, Utrecht, Netherlands

**Keywords:** cochlear implantation (CI), bilateral cochlear implantation, sequential bilateral cochlear implantation, sequential bilateral cochlear implant, cochlear implant, unilateral cochlear implant, unilateral cochlear implantation

## Abstract

**Introduction:** Previous studies have proven the effectiveness of bilateral cochlear implantation compared to unilateral cochlear implantation. In many of these studies the unilateral hearing situation was simulated by switching off one of the cochlear implants in bilateral cochlear implant users. In the current study we assess the accuracy of this test method. Does simulated unilateral hearing (switching off one cochlear implant) result in the same outcomes as real life unilateral hearing with one cochlear implant and a non-implanted contralateral ear?

**Study design:** We assessed the outcomes of one arm of a multicenter randomized controlled trial.

**Methods:** In the original trial, 38 postlingually deafened adults were randomly allocated to either simultaneous bilateral cochlear implantation or sequential bilateral cochlear implantation. In the current study we used the data of the sequentially implanted group (*n* = 19). The primary outcome was speech perception-in-noise from straight ahead. Secondary outcomes were speech perception-in-silence, speech intelligibility-in-noise from spatially separated sources and localization capabilities. A within-subjects design was used to compare the results of hearing with one cochlear implant and a non-implanted contralateral ear (1- and 2-year follow-up) with the results of switching off one cochlear implant after sequential bilateral implantation (3-year follow-up).

**Results:** We found no significant differences on any of the objective outcomes after 1-, 2-, or 3-year follow-up.

**Conclusion:** This study shows that simulating unilateral hearing by switching off one cochlear implant seems a reliable method to compare unilateral and bilateral hearing in bilaterally implanted patients.

**Clinical Trial Registration:** Dutch Trial Register NTR1722.

## Introduction

Cochlear implantation (CI) has become a widely applied intervention in the treatment of patients with severe to profound bilateral sensorineural hearing loss, who obtain limited benefit from conventional hearing aids.

Although many patients with a single cochlear implant achieve high levels of speech perception-in-silence, even the most successful cochlear implantees experience difficulty with speech perception-in-noise and localization capabilities (1, 2).

In 2009, our study group started a randomized controlled trial (RCT) concerning the effectiveness of simultaneous bilateral CI (simBiCI) compared with either (1) unilateral CI (UCI) (1, 3), or (2) sequential bilateral cochlear implantation (seqBiCI) (4). This RCT demonstrated a significant benefit of simBiCI compared with UCI after a 1- and 2-year follow-up period in everyday listening situations with speech and noise coming from different directions and for the ability to localize sounds.

Earlier (cohort) studies showed similar benefits of BiCI compared to UCI, however they used different methods to simulate the unilateral listening situation. In most of these studies, differences between bilateral and unilateral hearing were assessed using a within-subjects study design by switching off one cochlear implant in a group of bilaterally implanted patients and comparing the results with the bilateral listening situation (5–13).

Our hypothesis was that this simulated unilateral listening situation would not be representative for an actual UCI situation. The electrode in a patient with bilateral implants would have diminished residual hearing. On the other hand, patients with bilateral implants are used to listening with two ears in everyday life, while in patients with a unilateral cochlear implant, patients may not have used one ear for an extensive period of time.

We performed the current study to assess the reliability of switching off one cochlear implant as a method to simulate unilateral hearing in bilateral cochlear implant users.

## Materials and Methods

### Study Design and Participants

Data for the current study were collected as part of a multicenter RCT that compared simBiCI to seqBiCI (2–4).

This study was approved by the Human Ethics Committees of all participating centers (University Medical Centers of Utrecht, Maastricht, Nijmegen, Leiden and Groningen) (NL2466001808), registered in the Dutch Trial Register (NTR1722) and conducted according to the Declaration of Helsinki. Written informed consent was obtained from all participants.

**Figure 1:**
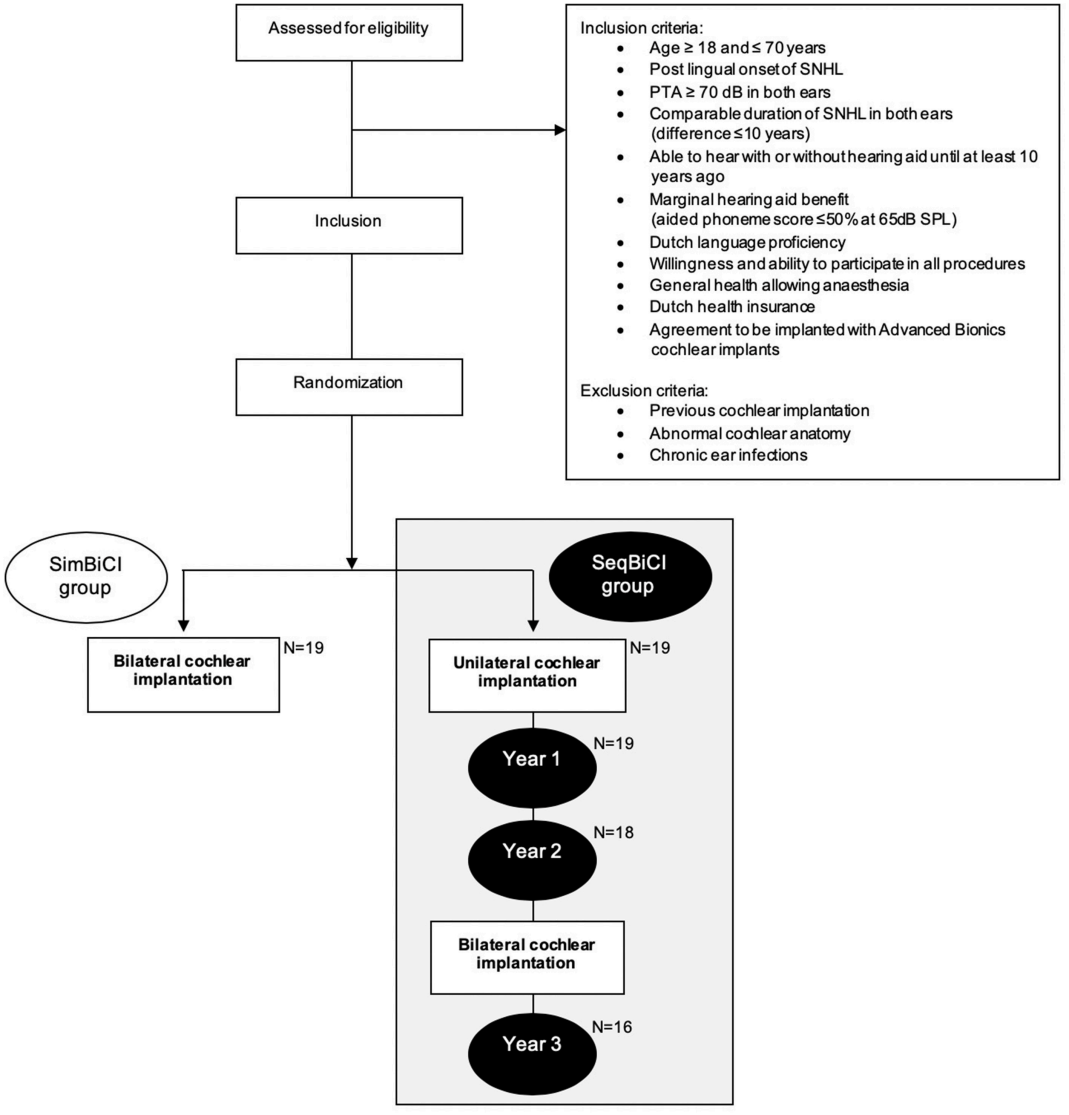
Study flowchart. SNHL, severe sensorineural hearing loss; PTA, pure tone audiometry (average of 500, 1,000, and 2,000 Hz); SimBiCI, simultaneous bilateral cochlear implantation; SeqBiCI, sequential bilateral cochlear implantation.

All participants eligible for CI were discussed in our cochlear implant team. Inclusion- and exclusion criteria were verified for each participant (Figure 1). After receiving informed consent and undergoing baseline hearing evaluations, patients were randomly allocated to either simBICI or seqBiCI (Figure 1). All participants were implanted with Advanced Bionics HiRes90K® (Advanced Bionics, Sylmar, CA, USA) and used Harmony processors with HiRes/HiRes120 processing strategies.

### Logistics

In the current study, we will focus on the first implanted side (CI1) in the seqBiCI group, by using a within-subjects design. Patients in this arm received their second cochlear implant 2-years after their first cochlear implant. Objective outcomes were measured after the first implantation at 1- and 2- years follow-up. After 2-years of follow-up, the patients received their second implant. In order to assess whether simulated unilateral hearing (switching off one cochlear implant) provides the same outcomes as real life unilateral hearing, we compared the data of this group 1- year after unilateral implantation (1-year follow-up) with the situation after bilateral implantation (3-year follow-up), in which we switched off the second cochlear implant (CI2). As a sensitivity analysis, to correct for a possible learning effect with the first implanted ear, we also compared the unilateral 2-year follow-up data with the simulated unilateral 3-year data (switching off the second cochlear implant).

### Outcome Measures

The primary outcome was speech perception-in-noise from straight ahead, measured with the Utrecht-Sentence Test with Adaptive Randomized Roving levels resulting in a speech reception threshold in noise (SRTn). A lower threshold value reflects better speech perception. A SRTn of 30 dB was considered speech perception in relative silence and was used as a cut-off point for all scores above 30 dB.

The other outcomes were (1) speech perception-in-silence, (2) speech intelligibility-in-noise from spatially separated sources (SISSS), and (3) localization capabilities. All these objective tests were conducted using the AB-York Crescent of Sound set-up. In previous articles of our study group more detailed information was presented about the test procedures and setup.

Speech perception in silence was measured using the standard Dutch consonant-nucleus-consonant (CNC) test.

In the SISSS, in which the outcome is also expressed as an SRTn, sentences were presented from 60° azimuth to the left of the patient and noise from 60° azimuth to the right of the patient (S-60 N+60) or vice versa (S+60 N-60). When sounds come from different directions, participants usually have a best performing situation and a worst performing situation. In current study, in which we evaluate the unilateral group or situation, speech presented to the cochlear implant side was indicated as the best performing situation. If speech was presented to the contralateral ear and noise to the cochlear implant side we indicated this as the worst performing situation.

For the localization test, participants were instructed to face the loudspeaker in front of them during the entire procedure. Thirty phrases were presented randomly at 60, 65, or 70 dB SPL from one of the loudspeakers. The results were percentage correct responses in three localization conditions: 15° angle azimuth between 5 loudspeakers, 30° angle azimuth between 5 loudspeakers, and 60° angle azimuth between 3 loudspeakers.

### Data Collection and Statistical Analysis

All gathered data were double-checked by two independent persons who did not have any further connections to the otorhinolaryngology department.

In order to compare baseline characteristics, means or medians were reported depending on normality of data. We used the Student *t*-test for numeric normally distributed data, the Wilcoxon signed rank test for non-normally distributed data and the chi-square test for ordinal data.

## Results

### Patient Characteristics and Missing Data

Between December 2009 and September 2012, a total of 19 participants were included in the seqBiCI group. Figure 1 shows a flowchart of the study. Baseline characteristics are described in Table 1.

**Table 1:** Baseline characteristics.

	**SeqBiCI (*n* = 19)**
Gender male:female	11:8
Age in years at CI	52.5 (12.5) [26–67]
Age start severe hearing loss AD	30.5 (20.1) [3–55]
Age start severe hearing loss AS	30.6 (19.8) [3–55]
PTA AD (dB)	106 (12) [78–125]
PTA AS (dB)	108 (13) [83–127]
Maximum phoneme score (%)	46.2 (20.4) [0–80]
**Treatment hospital**	
Utrecht	11
Maastricht	4
Nijmegen	2
Leiden	1
Groningen	1
**Hearing aid use before CI**	
Yes	19
No	0
**Cause of deafness**	
Hereditary	7
Unknown and progressive	9
Sudden Deafness	0
Head trauma	0
Meningitis	2
Rhesus antagonism	1
Sound exposure	0

*Mean (standard deviation) [range]. CI, cochlear implantation; AD, auris dextra; AS, auris sinistra; PTA, pure tone audiometry (average of 500, 1,000, and 2,000 Hz)*.

During the second and third year, two participants in the seqBiCI group withdrew because of personal reasons. Another participant was excluded because of poor results with the first implant and low expectations after sequential implantation owing to central deafness caused by rhesus antagonism (Figure 1).

### Objective Results

We found no significant differences between simulated unilateral hearing (switching off one cochlear implant) 1-year after seqBiCI and real life unilateral hearing 1- and 2-years after UCI. The results of all outcomes separately are presented in Table 2.

**Table 2:** Objective outcomes evaluating simulated and actual unilateral hearing in sequentially implanted cochlear implant users.

**Objective outcomes**	**Year 1 vs. year 3**			**Year 2 vs. year 3**		
	**Actual unilateral hearing**	**Simulated unilateral hearing (switching off CI2)**	*p-*value^*^	**Actual unilateral hearing**	**Simulated unilateral hearing (switching off CI2)**	*p-*value^*^
	***n* = 19**	***n* = 16**		***n* = 18**	***n* = 16**	
**Speech-in-noise both from straight ahead** (SRTn in dB)	10.6 (1.6–30.0)	10.0 (3.4–30.0)	*NS*	8.9 (2.2–30.0)	10.0 (3.4–30.0)	*NS*
**Phoneme score in silence** (CNC in %)	88.0 (64.0–98.0)	86.5 (42.0–98.0)	*NS*	85.0 (52.0–98.0)	86.5 (42.0–98.0)	*NS*
**Speech-in-noise from spatially separated sources**						
Best performing situation (SRTn in dB)	3.1 (−5.9–30.0)	5.1 (−5.3–30.0)	*NS*	3.8 (−5.6–30.0)	5.1 (−5.3–11.6)	*NS*
Worst performing situation (SRTn in dB)	16.9 (6.3–30.0)	18.4 (8.1–30.0)	*NS*	19.1 (4.1–30.0)	18.4 (8.1–30.0)	*NS*
**Localization of sounds**						
60° (% correct)	40.0 (33.3–56.7)	41.7 (30.0–63.3)	*NS*	35.0 (23.3–53.3)	41.7 (30.0–63.3)	*NS*
30° (% correct)	23.3 (13.3–33.3)	23.3 (13.3–46.7)	*NS*	20.0 (13.3–33.3)	23.3 (13.3–46.7)	*NS*
15° (% correct)	20.0 (16.7–40.0)	20.0 (13.3–33.3)	*NS*	20.0 (10.0–40.0)	20.0 (13.3–33.3)	*NS*

*Data are presented as median (range)*.

* *Wilcoxon signed rank test. SRTn, speech reception threshold in noise; CNC, consonant-nucleus-consonant words; CI2, second implanted ear in sequentially bilateral cochlear implant users; NS, not significant (p > 0.05)*.

## Discussion

### Key Findings

In order to assess methodological issues with simulation of cochlear implant use, in present study we assessed whether simulated unilateral hearing (switching off one cochlear implant) provides the same outcomes as real life unilateral hearing.

Binaural hearing has been proven to be superior to unilateral hearing with regard to speech perception in noise and sound localization (2, 3, 14–17). In previous studies, our study group concluded that there is a significant benefit of hearing with two implants compared to hearing with one implant in everyday listening situations in which speech and noise come from different directions (2, 3). Furthermore, bilaterally implanted patients are able to localize sounds, which is impossible for unilaterally implanted patients. Switching off one cochlear implant is an often-used method to assess the differences between uni- and bilateral hearing in bilateral implantees (5–13).

We assessed if this is a reliable test method.

However, the current study demonstrated similar results after UCI and hearing with one cochlear implant switched off after seqBiCI on speech perception-in-silence, speech intelligibility-in-noise and localization tests.

### Strengths and Limitations

This is the first study that reports whether simulated unilateral hearing (switching off one cochlear implant) provides the same results as real life unilateral hearing.

A strength of our study is that we used a prospective within-subjects study design. All data were collected at fixed moments. Secondly, by measuring after a follow-up of at least 1-year after implantation, it was safe to assume that patients were used to their implants and that we had corrected for a possible learning effect. Furthermore, at time of inclusion, all patients suffered from profound sensorineural hearing loss [pure-tone average of greater than 90 dB (threshold at 0.5, 1, 2, 3 kHz)]. Because of the profound hearing loss in the contralateral ear (second implanted ear) patients were not used to listening with two ears after the first implantation. Therefore, the situation before the second implantation can be considered as actual unilateral hearing.

As this study was based on a secondary analysis from a larger RCT, a power analysis for the present study was not performed and the study may be underpowered.

## Conclusion

We found no significant differences between simulated unilateral hearing (switching off one cochlear implant) and real life unilateral hearing. This study shows that simulating unilateral hearing by switching off one cochlear implant seems a reliable method to compare unilateral and bilateral hearing in bilaterally implanted patients.

## Ethics Statement

This study was carried out in accordance with the recommendations of the medical ethics committee (MEC) of the Academic Medical Center (AMC) with written informed consent from all subjects in accordance with the Declaration of Helsinki. The protocol was approved by the MEC AMC. This study was also registered in the Dutch Trial Register (NTR1722).

## Author Contributions

All authors have made substantial contributions to the paper. AvZ collected parts of the data, analyzed the data, and wrote the paper. YS designed the study, recruited and included participants, collected parts of the data, and critically revised the paper. VK collected parts of the data and critically revised the paper. GvZ provided audiometric advice and critically revised the paper. RS recruited and included participants and critically revised the paper. IS provided advice and was involved in the methodology for present study, statistical analysis and co-authored the paper.

### Conflict of Interest Statement

The authors declare that the research was conducted in the absence of any commercial or financial relationships that could be construed as a potential conflict of interest.
